# The Role of Disaccharidase Deficiencies in Functional Abdominal Pain Disorders—A Narrative Review

**DOI:** 10.3390/nu10121835

**Published:** 2018-11-29

**Authors:** Mora V. Puertolas, Amanda C. Fifi

**Affiliations:** 1 Department of Pediatrics, Holtz Children’s Hospital, University of Miami Miller School of Medicine/Jackson Memorial Medical Center, 1611 NW 12th Ave, Miami, FL 33136, USA; 2 Department of Pediatric Gastroenterology, Hepatology and Nutrition, University of Miami Miller School of Medicine, 1601 NW 12th Ave, Miami, FL 33137, USA; afifi@med.miami.edu

**Keywords:** sucrase, maltase, isomaltase, irritable bowel syndrome, functional abdominal pain, functional dyspepsia

## Abstract

Disaccharidase deficiencies are reportedly underdiagnosed in pediatric populations. Though typically thought to cause diarrheal disease, they can also be a cause of abdominal pain and dyspepsia, and patients diagnosed with these functional disorders may actually have associated enzyme deficiencies. While the effects of lactose deficiency have been widely studied, sucrase, maltase, and isomaltase are less frequently considered when approaching a patient with an apparent functional abdominal pain disorder. This review seeks to provide an up-to-date narrative on the current scientific literature on the possible role of sucrase, maltase, and isomaltase deficiency in pediatric functional gastrointestinal disorders.

## 1. Introduction

Disaccharidases are glycoside hydrolase enzymes found in the intestinal brush border that are responsible for the breakdown of disaccharides into monosaccharides. A congenital or acquired deficiency of these important enzymes results in an excess of carbohydrate substrate in the small bowel, which increases osmotic load leading to increased luminal fluid. Excessive fermentable substrate also increases gas production in the small bowel. The resultant small bowel distension can lead to feelings of early satiety and bloating in patients thought to have functional dyspepsia and irritable bowel syndrome and to abdominal pain, especially in patients with visceral hypersensitivity [1].

**Figure 1:**
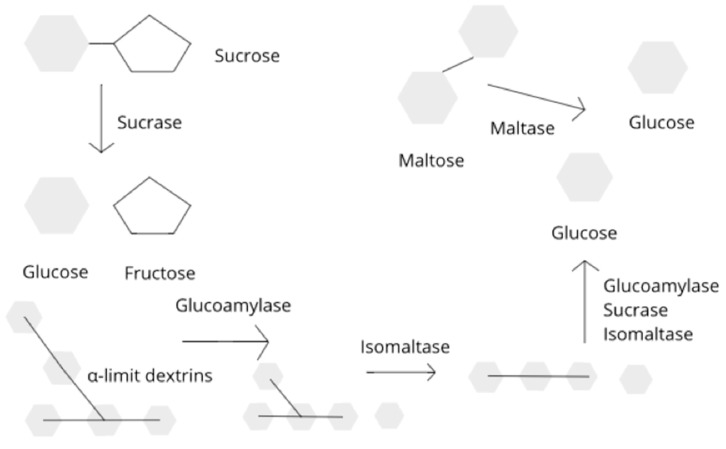
Schematic representation of the enzymatic degradation of starches and disaccharides into monosaccharides.

Sucrase is responsible for the breakdown of sucrose to glucose and fructose and plays a major role in starch oligomer digestion to glucose. Sucrose is common in the Western diet and found in a variety of foods including fresh fruits, such as peaches and bananas, and in desserts, such as cookies and chocolates, along with many popular drinks [2]. Isomaltase splits α-1,6 linkages in the branched poly- and oligosaccharides, products of amylase digestion that cannot be digested by amylase or maltase [3]. Glucoamylase works in the brush border in conjunction with amylase to further hydrolyze oligosaccharides into glucose [4]. Isomaltase works in conjunction with sucrase as part of the brush border enzyme sucrase-isomaltase [5]. Maltase breaks down the disaccharide maltose to the simple sugar glucose and is produced by the cells that line the small intestine [6]. Maltose is found in caramelized foods and is a product of foods containing starch. A schematic representation of the enzymatic degradation of starches and disaccharides is outlined in Figure 1. Deficiencies in any one of these enzymes can result in intolerance to these everyday foods, leading to dyspepsia, bloating, and abdominal pain that can mimic the symptoms of functional gastrointestinal disorders (FGIDs) in the pediatric population [7]. Rome IV criteria characterize FGIDs by symptomatology in an attempt to make it easier for clinicians to diagnose and treat these conditions [8]. FGIDs include a wide range of conditions related to the gastrointestinal tract not caused by structural or biochemical abnormalities. These conditions occur with frequency in children of all ages. It is believed that a subset of FGID patients actually have associated disaccharidase deficiencies that, if identified, can lead to better treatment outcomes for the affected patients [9]. Disaccharidase deficiencies can be congenital or acquired [10], thus leading to further confusion in appropriate diagnosis and subsequent management of these patients.

Although initially thought to be rare, many genes are associated with disaccharidase deficiency [11]. Congenital sucrase-isomaltase deficiency (CSID), also known as genetic sucrase-isomaltase deficiency (GSID), is diagnosed by genetic testing and consists of 26 different mutations, of which four are the most frequent [12]. This condition is autosomal recessive, but presentation can be highly variable depending on the degree of the mutation. The prevalence of CSID varies by population. CSID is estimated to occur in 1 in 5000 people of European descent, however, in the native populations of Greenland, Alaska, and Canada, it is believed to be much more common, with approximately 1 in 20 people having a genetic mutation [12].

## 2. Disaccharidase Deficiencies in Patients with Gastrointestinal Symptoms that Mimic Functional Abdominal Disorders

Abdominal pain is the most commonly described symptom in pediatric patients with disaccharidase deficiency (94%), followed by diarrhea (46%), and nausea/dyspepsia (40%), as shown in Table 1 [13]. Studies have also found constipation to be a common presenting complaint [1]. As such, disaccharidase deficiency should be considered as a possible diagnosis when evaluating a child with symptoms consistent with a FGID. Table 2 outlines the studies reviewed that found an association between FGIDs and disaccharidase deficiencies.

El-Chammas et al. (2017) evaluated 203 pediatric patients presenting specifically with abdominal pain who underwent biopsies to test for disaccharidase activity [9]. A large proportion (49%) of these patients had a least one disaccharidase deficiency. Additionally, they found 36.5% had low lactase activity and 21% had low sucrase activity, suggesting a correlation between abdominal pain and disaccharidase deficiencies. However, El-Chammas et al. could not find a correlation between clinical features, namely epigastric pain, vomiting, and diarrhea, and the specific disaccharidase deficiency identified on testing [9]. Furthermore, Chumpitazi et al. could not find a correlation between the disaccharidase level and the severity of symptoms [14]. This makes it difficult to predict the subset of patients with functional abdominal complaints in whom various disaccharidase testing would be useful. Thus, a clinical awareness of these conditions is important when approaching a child with a presumed FGID.

In a pediatric study of 129 patients ranging in age from 4.1 to 16.1 years with chronic dyspepsia undergoing endoscopy, Chumpitazi et al. (2018) found 47.5% of these patients had an underlying disaccharidase deficiency [14]. Nichols et al. (2012) reviewed 27,875 pediatric endoscopic biopsies sent for disaccharidase testing at a reference laboratory. Forty-six percent of samples had a deficiency of at least one of the four enzyme activities tested. While lactase deficiency was the most common, total sucrase deficiency was found in 9.3% of samples tested [21]. In another study of 938 patients undergoing upper endoscopy with disaccharidase testing, Cohen et al. (2018) found sucrase deficiency (7.3%) to be the most common entity after lactase deficiency, with maltase deficiency being the least common (0.8%). Of all patients with lactase deficiency, 39% also had sucrase deficiency and out of all patients with sucrase deficiency 67% also had lactase deficiency. Pan-deficiency was found in 24 of the patients in whom all four enzymes were tested (9.9%) [13]. Finally, a larger metanalysis of 30 pediatric studies examining 34,753 disaccharide assays found the proportion of lactase deficiency to be 39.2%, maltase deficiency 12.6%, sucrase deficiency 9.0%, and palatinase (isomaltase) deficiency 9.1% [22].

Cohen and Oloyede (2018) found that despite the apparent frequency of disaccharidase deficiencies, many pediatric gastroenterologists were not considering this diagnosis at the time of endoscopy [11]. Of the 3235 patients undergoing upper endoscopy, only 963 patients (17.9%) underwent disaccharidase analysis and, of these, deficiency of sucrase and primary sucrase-isomaltase was identified in 7.6% and 3.5%, respectively. They also found that the number of pediatric gastroenterologists who routinely considered these conditions by sending duodenal biopsies in patients with complaints of dyspepsia, emesis, abdominal pain, or diarrhea varied widely, from 1.6% to 64.5%. A survey of participating physicians showed that the variability could be explained by their practice style, with physicians being more likely to obtain biopsies in cases of unexplained abdominal pain, especially when diarrhea or bloating were also present. The difference in practice was not explained by previous training, practice duration, or knowledge of disaccharidase deficiencies. This suggests that guidelines for testing patients presenting with abdominal complaints should be reviewed and that testing for these deficiencies should be considered in certain clinical scenarios, for example, in patients with intractable symptoms.

CSID can also present with symptoms similar to those of IBS, such as diarrhea, bloating, and abdominal pain [12]. Henstrom et al. (2018) conducted a study investigating genetic variations in the SI gene of eight Caucasian patients—seven familial subjects with severe post-prandial IBS-D (Irritable Bowel Syndrome - Diarrhea Subtype) and one asymptomatic relative—and screened for CSID mutations in 1887 cases (mean age: 40.3 years) of IBS (1031) and controls (856). They found that patients with IBS-D and IBS-M (Irritable Bowel Syndrome - Mixed Subtype) were more likely to have genetic variations in the SI gene [15]. Heterozygote carries of the CSID mutation had an almost two-fold increased risk of IBS (*p* = 0.074; *OR* = 1.84). Additionally, Garcia-Etxebarria et al. (2018) studied the genotype of 2207 IBS patients and found that, when compared with a matched reference population from ExAC (Exome Aggregation Consortium), most rare sucrase-isomaltase pathogenic variants were identified in IBS patients and further analysis showed that these variants increased the risk of IBS [16].

## 3. Disaccharidase Deficiencies Can Coexist with Organic Diseases

In addition to being identified as a cause or contributing factor of FGIDs, disaccharidase deficiencies can also coexist with other gastrointestinal conditions [23]. While treatment of the underlying condition is necessary, recognizing and treating an associated enzyme deficiency can be a temporizing measure to allow symptom relief in the interim or may explain persistence of symptoms after the initiation of treatment. O’Grady et al. (1984) reported an association between inflammation on histology with lactase and sucrase deficiency [24]. Heitlinger et al. (1991) found an inverse correlation between lactase and maltase levels and inflammation on biopsy. In a review done on seven studies of adult patients with celiac disease, they found decreased disaccharidase levels in patients with more severe inflammation when compared to those with celiac without inflammation. Mean sucrase levels were 18.3 mmol/min/g of protein and 45.14 mmol/min/g of protein, respectively [17]. This was also true for lactase and maltase levels. In a retrospective study spanning eleven years, Sun et al. (2015) reviewed the histologic specimens from ten pediatric patients, aged 5 to 18 years, who met diagnostic criteria for lymphocytic colitis. The authors identified a statistically significant correlation between lymphocytic colitis and lactase deficiency, indicating that disaccharidase deficiencies could be an additional mechanism of diarrhea in this group of patients [25].

## 4. Diagnosis and Management of Disaccharidase Deficiency

There are various methods for diagnosing disaccharidase deficiencies. Breath tests, genetic tests, and even an oral trial of the various formulations of the enzyme have all been used with varying success [18,19]. The Dahlqvist method for assessing disaccharidase activity uses an intestinal homogenate, collected by biopsy, that is incubated with various disaccharide substrates [26]. The disaccharidases most commonly assessed include: lactase, sucrase, palatinase (isomaltase), and maltase. In modern day disaccharidase testing, this method, with some modifications, is still considered the gold standard, with purported normal levels shown in Table 3 [27]. However, true reference ranges for normal enzyme activities are difficult to determine, given that biopsy samples, obtained by an invasive endoscopy, represent a skewed population and not a true “normal” reference population. Hackenmueller et al. (2016) employed the Hoffman method to ascertain a more reliable reference range and reported the cutoffs to be lactase 5 U/g protein, maltase 105 U/g protein, palatinase (isomaltase) 9 U/g protein, and sucrase 26 U/g protein [28].

While universal testing of patients presenting with functional gastrointestinal complaints is not always recommended, the possibility of CSID should at least be considered in patients with functional abdominal complaints associated with diet, patients with persistent symptoms despite employing other treatment modalities, and/or those with risk factors, such as known CSID mutations in family members. The invasiveness of the Dahlqvist test, given that it requires endoscopic samples, hinders its use as a routine diagnostic tool in pediatric patients and thus alternative means of testing have been studied. Robayo-Torres et al. (2018) studied eleven patients with biopsy-proven CSID and six controls and found that C-sucrose breath testing correlated with sucrase activity on biopsy, making it a good diagnostic tool [29]. All eleven patients with CSID had impaired starch digestion, as evidenced by their breath test, making the test 100% sensitive in this small sample of patients. In a study of eleven adult patients with FGIDs and eleven controls, Opekun et al. (2016) found that eight of the patients with FGIDs had decreased sucrose digestion, as indicated by a biphasic C14- sucrose glucose breath test (14C-S/GBT) less than 85% the normal level after 60–75 min of disaccharide ingestion [18]. The studies by Henstrom and Garcia-Etxebarria proposed that genetic testing may be a less invasive and promising method of identifying mutations in the sucrase-isomaltase gene in patients with IBS and therefore allows for a tailored approach to therapy in this subset of patients [15,16].

There is limited information on the treatment of children with FGIDs that are subsequently diagnosed with disaccharidase deficiencies. Despite studies showing deficits of disaccharidases in a subset of children with FGIDs, presently there is not sufficient evidence that the treatment with enzyme replacement will improve symptoms. A non-randomized longitudinal study by Puntis et al. (2015) suggests that enzyme replacement may be helpful [19]. Although their sample size consisted of only six patients between the ages of 0.5 and 2.5, all of whom presented with diarrhea, all six patients had resolution of their diarrhea upon institution of enzyme replacement and all had a return of their diarrhea when enzyme replacement was discontinued. They further postulated that a trial of oral sucrase could even potentially be used as a diagnostic tool, with patients found to have relief of their symptoms after a trial of oral sucrase upon being diagnosed as sucrase deficient. Treem et al. investigated 28 children (aged 5 months to 11 years) in a randomized, double-blinded clinical trial in which they underwent breath testing after receiving placebo, sacrosidase, and sacrosidase plus milk [20]. Patients who received sacrosidase or sacrosidase plus milk had significantly decreased breath H2 excretion when compared to placebo. In the second phase of the study, subjects received four multidose sacrosidase treatments of different strengths for 10 days with higher concentrations of sacrosidase found to be associated with fewer stools (0.001) and fewer symptoms of gas, abdominal cramps, or bloating, but no difference was found with vomiting. These small studies emphasize the need for larger, randomized, placebo-controlled studies to assess the usefulness of oral sucrase formulations in treating symptoms of abdominal pain, bloating, and diarrhea in these patients and the use of oral sucrase formulations as a diagnostic test. Notably, enzyme replacement therapy cannot be used to distinguish between congenital disaccharidase deficiency and acquired disaccharidase deficiency for diagnostic purposes because the underlying pathology in both patient groups is the lack of enzymes at the level of the enterocytes.

Treatment with dietary restriction of sucrose and starch has shown mixed results, with 60–75% of patients adhering to the diet with persistent diarrhea and/or abdominal pain. Additionally, compliance to diet was found to be poor at 50% [19]. Chumpitazi et al. conducted a double-blind crossover trial of 33 IBS children, fulfilling the IBS Rome III criteria, with a mean age of 11.5 ± 3.0 years [14]. Children were randomized to receive a low FODMAPs (Fermentable Oligosaccharides, Disaccharides, Monosaccharides and Polyols) diet and then crossed over to a typical American childhood diet (TACD). Abdominal pain was reported less frequently among children on low FODMAPs diet compared to TACD (1.1 ± 0.2 vs. 1.7 ± 0.4 pain episodes per day, respectively; *p* < 0.05). This reiterates the role of dietary carbohydrates, typically in the TACD, in the pathophysiology of some FGIDs.

## 5. Conclusions

This review suggests that disaccharidase deficiencies should be considered in the differential for children presenting with abdominal pain, dyspepsia, diarrhea, and even constipation presumed to be functional in nature [13]. Several studies have demonstrated that disaccharidase deficiencies are more common in children than previously thought [11] and many disaccharidase deficiencies can coexist, like lactase and sucrase deficiencies [9]. The variable presentations of CSID, also called GSID, can also delay diagnosis until teenage years and even adulthood. Furthermore, disaccharidase deficiencies can coexist with other gastrointestinal disorders, such as IBD, celiac disease, and lymphocytic colitis [25]. It is important to note that the prevalence of disaccharidase deficiency in many study populations is biased, as the children that were tested were being investigated for a gastrointestinal complaint, thus it may not represent the prevalence of their deficiency in the pediatric population at large. However, obvious ethical reasons limit our ability to obtain sample biopsies from otherwise healthy children. Less invasive studies, such as genetic testing and breath testing, of otherwise healthy subjects may help elucidate more accurate prevalence in the pediatric population.

Despite available evidence, many pediatric gastroenterologists do not test for these deficiencies [11], potentially leading to a delay in diagnosis and treatment. Guidelines for incorporating enzyme assay testing for certain pediatric patients that are undergoing endoscopy for abdominal pain, dyspepsia, or diarrhea could lead to a change in practice and a more timely diagnosis. Diagnosis can also be made by breath testing [28] and, in future, genetic testing [12] may be more widely available to discover the various mutations responsible for these deficiencies. Some studies even suggest an oral trial of the suspected deficient enzyme may be used as a diagnostic tool [19]. Further research is needed to develop a protocol to determine which subset of patients with seemingly functional disorders should be tested and subsequently treated. A focused treatment approach in this group of patients may lead to better outcomes and improved quality of life. Currently the use of enzyme replacement, with or without dietary restriction, holds the most promise for treatment and further large, randomized, placebo-controlled studies are warranted to confirm their benefits.

## Figures and Tables

**Table 1:** Most common presenting complaints of disaccharidase deficiency.

Most Common Presenting Complaints of Disaccharidase Deficiency
Abdominal pain 94%
Diarrhea 46%
Nausea/Dyspepsia 40%

**Table 2:** Studies reviewed that found a relationship with possible functional gastrointestinal disorders (FGIDs) and disaccharidase deficiencies.

Study Authors	Number of Participants	Age of Participants in Years	Presenting Complaint/Diagnosis	Findings
El-Chammas et al. [9]	203	Not provided	Abdominal pain	49% of patients presenting with abdominal pain had a disaccharidase deficiency
Cohen and Oloyede [11]	963	4.6–6.1	Abdominal pain, diarrhea, constipation, nausea, poor weight gain, flatulence	7.6% had sucrase deficiency and 3.5% had sucrase-isomaltase deficiency
Cohen et al. [13]	963	4.6–14.1	Database of patients having EGD who also had disaccharidase testing	Sucrase deficiency (7.35%) was most common after lactase; maltase deficiency was least common (0.8%)
Chumpitazi et al. [14]	129	4.1–16.1	Chronic dyspepsia	47.5% had underlying disaccharidase deficiency
Henstrom et al. [15]	1887	Mean age: 40.3	IBS-like symptoms	Patients more likely to have mutations in SI gene
Garcia-Etxebarria et al. [16]	2207	Not provided	598 IBS with IBS-C, 952 IBS with IBS-D, 503 IBS with alternating constipation and diarrhea, and 154 un-subtyped IBS	Sucrase-isomaltase variants had a slightly higher prevalence (3.995%) in the IBS-D and IBS-C groups
Heitlinger et al. [17]	798	Adult	Gastrointestinal symptoms requiring capsule or endoscopic biopsy	Diminished disaccharidase activity in mucosal injury inversely correlated with degree of injury
Opekun et al. [18]	8	Not provided	FGIDs	Breath testing was 85% of normal after 60–75 min of disaccharide ingestion
Puntis and Zamvar [19]	6	0.5–2.5	Diarrhea	Symptom resolution with enzyme replacement and return of diarrhea when enzyme replacement discontinued
Treem [20]	28	0.5–11	CSID	Sacrosidase resulted in symptom improvement

EGD: esophagogastroduodenoscopy; IBS: irritable bowel syndrome; IBS-C: irritable bowel syndrome—constipation subtype; IBS−D: irritable bowel syndrome—diarrhea subtype; FGIDs: functional gastrointestinal disorders; CSID: congenital sucrase-isomaltase deficiency.

**Table 3:** Historical cutoff activities that are used to identify a disaccharidase deficiency.

Values for Normal Enzyme Levels	U/g
Lactase	<15
Maltase	<100
Palatinase	<5
Sucrase	<25
